# Collagen/kerateine multi-protein hydrogels as a thermally stable extracellular matrix for 3D *in vitro* models

**DOI:** 10.1080/02656736.2021.1930202

**Published:** 2021

**Authors:** Kameel Zuniga, Manasa Gadde, Jacob Scheftel, Kris Senecal, Erik Cressman, Mark Van Dyke, Marissa Nichole Rylander

**Affiliations:** a Department of Biomedical Engineering, The University of Texas at Austin, Austin, TX, USA;; b Natick Soldier Center, U.S. Army Soldier and Biological Chemical Command, Natick, MA, USA;; c Department of Interventional Radiology, The University of Texas MD Anderson Cancer Center, Houston, TX, USA;; d College of Biomedical Engineering, The University of Arizona, Tucson, AZ, USA;; e Department of Mechanical Engineering, The University of Texas at Austin, Austin, TX, USA

**Keywords:** Hydrogel, collagen, kerateine, hyperthermia, biomimetic engineering

## Abstract

**Objective:**

To determine whether the addition of kerateine (reduced keratin) in rat tail collagen type I hydrogels increases thermal stability and changes material properties and supports cell growth for use in cellular hyperthermia studies for tumor treatment.

**Methods:**

Collagen type I extracted from rat tail tendon was combined with kerateine extracted from human hair fibers. Thermal, mechanical, and biocompatibility properties and cell behavior was assessed and compared to 100% collagen type I hydrogels to demonstrate their utility as a tissue model for 3D *in vitro* testing.

**Results:**

A combination (i.e., containing both collagen ‘C/KNT’) hydrogel was more thermally stable than pure collagen hydrogels and resisted thermal degradation when incubated at a hyperthermic temperature of 47°C for heating durations up to 60 min with a higher melting temperature measured by DSC. An increase in the storage modulus was only observed with an increased collagen concentration rather than an increased KTN concentration; however, a change in ECM structure was observed with greater fiber alignment and width with an increase in KTN concentration. The C/KTN hydrogels, specifically 50/50 C/KTN hydrogels, also supported the growth and of fibroblasts and MDA-MB-231 breast cancer cells similar to those seeded in 100% collagen hydrogels.

**Conclusion:**

This multi-protein C/KTN hydrogel shows promise for future studies involving thermal stress studies without compromising the 3D ECM environment or cell growth.

## 1. Introduction

Hyperthermia has profound effects on biology in living systems [1–3]. In medical applications, therapeutic hyperthermia ranges from mild (generally up to 43 °C) to very high temperatures up to 47 °C as seen with thermal ablation through various methods [4]. Even with ablation, however, mild hyperthermia conditions transiently occur at the margins of a treated zone [5]. Studies of thermal stress are commonly performed with 2D cell culture, yet 3D systems would provide higher fidelity models for thermal biology studies by enabling measurement of both spatial and temporal responses in a more physiologically representative context. Collagen type I has been effectively utilized as a representative extracellular matrix (ECM) for 3D *in vitro* platforms stemming from the fact that collagen comprises approximately 90% of proteins in human connective tissues. It is readily available and can effectively be used as a model of human ECM [6–14]. Rylander and coworkers have conducted extensive characterization of collagen hydrogels based on collagen concentration, polymerization time, pH and temperature and determined how changing these parameters influences collagen porosity, mechanical properties and diffusivity of particles representative of drug compounds [15]. Kutschka et al. and Doyle et al. also demonstrated that changes in collagen concentrations also led to changes in the hydrogel stiffness, which allowed the control of cell proliferation of cardiomyoblasts and fibroblasts, respectively [16,17]. The corresponding tumor cell response and angiogenesis have been measured in collagen type I hydrogels with high collagen density (8 mg/ml) to mimic the elevated stiffness of tumors used in previous *in vitro* tumor models [18–21]. Healthy tissue is significantly less stiff; most studies of healthy tissue using *in vitro* models use collagen concentrations ranging from 1 to 4 mg/ml [22–26].

Although collagen type I has many benefits for creating 3D *in vitro* models, it cannot be used to study cell behavior in response to thermal changes, which is essential for planning hyperthermia treatments [27–29], assessment of response to burn injury [30,31] and thermal preconditioning studies to enable determination of appropriate parameters [32,33]. Thermal preconditioning of tissues can be crucial in wound healing, including bone and skin tissue regeneration [34,35]. In addition, the combination of immunotherapy with hyperthermia treatment has been shown to generate heat shock proteins that in turn increase tumor antigen presentation and dendritic cell migration, improving antitumor immune responses [36]. Collagen has a low denaturing temperature of approximately 38–45 °C and begins melting at even lower temperatures [37–39]. In contrast, skin, tendons, tumors and other biological tissues have a higher denaturing temperature reaching up to 60 °C [40] because tissue contains other proteins besides collagen, including glycosaminoglycans, elastins and keratin.

Many hyperthermia experiments use *in vitro* cell monolayers as their model system because of the low melting temperature of collagen hydrogels. This is not an accurate representation of heat transfer through an organ or tissue because diffusion properties and cell response to stimuli differ in a 3D environment [30,41,42]. The use of *ex vivo* tissue addresses the spatial limitation of cell monolayers, but these models can be cost-prohibitive and may not closely resemble living human tissue if isolated from non-human tissue. The viability and integrity of *ex vivo* tissues are also challenging to maintain for sufficient durations due to tissue collection and handling and culture conditions [43,44]. Non-native materials such as sodium alginate can have greater thermal stability, enabling measurement of cell response to hyperthermia in 3D [27,45]. However, alginate and other non-native materials do not include the necessary cell-binding motifs to target cell response and adhesion, inhibiting optimal cell growth [46–49]. Therefore, an ECM with greater thermal stability is needed that does not compromise the 3D spatial architecture needed to mimic native tissue and allow for cell–ECM interactions.

Previous studies on keratin’s thermal stability by Balaji et al. utilized keratin extracted from animal horn meal combined with collagen. This resulted in greater thermal stability with higher melting temperature (*T*_m_) measured by differential scanning calorimetry (DSC) [50]. However, this study lacked comparisons to standard collagen type I hydrogels without keratin, and extensive characterization was not performed for material properties and cell growth. Keratin shows excellent promise as a material for biological, thermal stress testing and has a higher *T*_m_ than collagen type I, falling between 120 and 150 °C when wet and 240 °C when dry [51–53]. The inclusion of keratin has biological relevance since it includes binding motifs to increase cell attachment, promoting cell growth, allowing the potential to be used for thermal stress studies without compromising the ECM [54–59].

Two forms of keratin exist based on how they are extracted: reductive and oxidative chemistries, resulting in kerateine (KTN) and keratose (KOS), respectively. KTN contains reduced cysteine residues that can form disulfide crosslinks [60]. KOS, however, is formed by protein oxidation in which disulfide linkages are converted to sulfonic acid groups, allowing greater solubility of the cortical proteins [61]. The disulfide bonds in KTN-based products mean that these biomaterials are more stable and degrade more slowly than their KOS-based counterparts. KTN results in a more stable hydrogel without the addition of an exogenous crosslinker [62] and increases in cell binding motifs from the addition of KTN [54,55].

In the present study, we explored how the addition of KTN to collagen type I 3D *in vitro* hydrogels could modulate the thermal and mechanical properties and cellular response, and thus its suitability for future investigation into the effect of thermal stress/treatments on cell behavior. We fabricated C/KTN hydrogels and measured the structural, mechanical, thermal and biological characteristics of different collagen to KTN ratios in the context of a 3D ECM. Cell behavior in response to thermal changes is vital for planning hyperthermia treatments [27–29], assessing burn injury and wound healing [30,31], and thermal preconditioning studies to induce heat shock proteins [32,33], and overall survival of healthy cells. Multiple studies creating 3D *in vitro* models mimicking healthy tissue have used collagen concentrations ranging from 2 to 4 mg/ml, specifically for human skin [23–26,63]. To mimic healthy tissue, 3 mg/ml hydrogels were made with different collagen to KTN ratios (50/50, 30/70, 20/80 C/KTN), seeded with fibroblasts that are found in all types of healthy tissue [64,65]. To mimic tumor tissue with a higher mechanical stiffness [22,66–68], we employed a 7 mg/ml collagen hydrogel similar to previous studies published by our lab with the same collagen to KTN ratios [15,23,64,69]. Hydrogel micro-scale architecture was observed through scanning electron microscopy (SEM), and mechanical properties were characterized through rheological measurements. Thermal properties of hydrogels were characterized by DSC and a thermal degradation assay. Cell response was characterized by measuring cell viability, metabolic activity, and live- and actin-staining to quantify live cell number and spreading (aspect ratio (AR)), respectively.

## 2. Materials and methods

### 2.1. Collagen extraction

Collagen type I was isolated from rat tail tendons in a 1 N HCl solution (Fisher Scientific, Hampton, NH) at approximate pH 2.0 and stirred for 16 h at room temperature. The solution was then transferred to 50 ml centrifuge tubes (Fisher Scientific, Hampton, NH) and centrifuged at 30,000×*g* (16,000 rpm) for 60 min at 4 °C to pellet the insoluble components. The supernatant was decanted from each tube and transferred into separate 50 ml centrifuge tubes, stored at −20 °C overnight, and freeze-dried for 48 h. Samples were dissolved in the appropriate amount of 0.01% glacial acetic acid (Fisher Scientific, Hampton, NH) for a final concentration of 6 or 14 mg/ml of collagen stock solution [64] to create 3 and 7 mg/ml collagen hydrogels, respectively.

### 2.2. Kerateine extraction

KTN was kindly donated by Dr. Mark Van Dyke and was extracted from human hair obtained from a commercial source. Briefly, human hair was washed, chopped into small pieces and soaked in a solution of 0.5 M thioglycolic acid (Sigma-Aldrich, St. Louis, MO) in deionized (DI) water for 12 h at 36 °C with gentle stirring [57]. Hair was then filtered from the liquid with a 500 μm sieve (W. S. Tyler, Mentor, OH), and the reducing solution retained. Free proteins were further extracted in excess 100 mM Tris base for 1 h, followed by DI water for 1 h with gentle shaking at 37 °C. Extracts were collected with the 500 μm sieve and combined with the reductant solution. This entire process was repeated one additional time, and all extracts were combined, centrifuged and filtered. The combined extracts were then purified and concentrated using a tangential flow filtration system that employed a 100 kDa cutoff membrane, frozen and lyophilized on a Labconco benchtop freeze-drying system under ambient conditions.

### 2.3. Cell culture

Normal human dermal fibroblasts (NHDFs, PromoCell, Heidelberg, Germany; isolated from the dermis of juvenile foreskin) were chosen to provide a representative healthy cell line for assessing response to the C/KTN hydrogels. The MDA-MB-231 breast cancer cell line (ATCC, Manassas, VA; triple-negative breast cancer cell line isolated from a metastatic mammary adenocarcinoma) is one example of the most commonly used in breast cancer research, employed in more than two-thirds of breast cancer studies and served as a representative cell line for assessment of tumor response [70]. Cells were seeded in T-75 Eppendorf HEPA-filtered flasks (Eppendorf, Hamburg, Germany). NHDFs were cultured in complete fibroblast media composed of fibroblast basal medium 2 (PromoCell, Heidelberg, Germany) supplemented with 2% fetal calf serum, 0.1% human fibroblast growth factor, 0.5% human insulin (PromoCell, Heidelberg, Germany) and 1% penicillin–streptomycin. MDA-MB-231 breast cancer cells were cultured in complete breast cancer cell media composed of DMEM/F12 50:50 basal medium (Sigma Aldrich, St. Louis, MO) supplemented with 10% fetal bovine serum (FBS) and 1% penicillin–streptomycin. All cells were maintained in 5% CO_2_ atmosphere at 37 °C in a sterile cell-culture incubator, and corresponding complete media was changed every two days. Cells were detached for hydrogel fabrication by washing with phosphate-buffered saline (PBS) and replaced with 0.04%/0.03% trypsin/ethylenediaminetetraacetic acid (EDTA) (PromoCell, Heidelberg, Germany) to NHDFs and 3 ml 0.25% trypsin, 0.1% EDTA in Hank’s balanced salt solution (HBSS) (Mediatech, Inc., Manassas, VA) to breast cancer cells, followed by incubation for 3 min. Trypsin/cell solution was neutralized with corresponding complete media, transferred to a 15 ml conical tube and centrifuged at 200×*g* for 3 min. The pellet was isolated and resuspended in 1 ml of complete media for cell counting with trypan blue.

### 2.4. Collagen/kerateine hydrogel fabrication

NHDFs cultured in a representative 3 mg/ml collagen solution were used to stimulate stiffness of normal tissue [15,22,25,26] and MDA-MB-231 breast cancer cells were cultured in a 7 mg/ml collagen solution corresponding to higher stiffness of tumor tissue [15,23,64,69]. One hundred percent collagen hydrogels were prepared from collagen stock solution of double the desired collagen concentration by neutralizing with 10× DMEM (Sigma-Aldrich, St. Louis, MO), 1× DMEM (Gibco, Gaithersburg, MD) and 1 N NaOH (Fisher Scientific, Hampton, NH) for a resulting pH of 7.4. 50/50, 30/70 and 20/80 w%/w% C/KTN hydrogels were prepared by combining stock collagen and KTN dissolved in a neutralizing buffer of equal volume. For example, to create a 3 mg/ml collagen hydrogel with an equal weight of KTN (50/50 C/KTN hydrogel) 6 mg/ml of KTN in neutralizing buffer was mixed with 6 mg/ml collagen stock solution. Table 1 describes the different hydrogels made with corresponding KTN concentration and total protein (collagen and keratin) concentration as w%/v%. Stock collagen and KTN/neutralizing buffer solution was combined at 1:1 v:v ratio and mixed thoroughly with a spatula and positive displacement pipette. For cell culture experiments, cells were embedded in the gel, resulting in a final concentration of 0.5 × 10^6^ (MDA-MB-321 breast cancer cells and NHDFs) or 0.1 × 10^6^ cells/ml (NHDFs only). Two seeding densities (0.1 and 0.5 × 10^6^/ml) were used for NHDFs because the initial seeding density of 0.5 × 10^6^/ml displayed no cell growth and proliferation over time. The hydrogel-cell solution was made by centrifuging and isolating the appropriate number of cells, adding the hydrogel mixture directly to the cell pellet, and mixed thoroughly with a positive displacement pipette. Hydrogel solution was added to cell culture-treated 96, 48 or 24 well-plates and incubated at 37 °C for 40 min to allow for polymerization. C/KTN hydrogels were cultured in media and changed every two days.

### 2.5. Scanning electron microscopy and analysis of ECM structure

Scanning electron microscopy was utilized to characterize the ECM-like structure of hydrogels, including the porosity, fiber orientation and fiber width, to determine whether the different ratios of C/KTN gave rise to different structures. Polymerized acellular hydrogels in 96 well-plates were fixed with aldehyde solution overnight, made from 0.2 M cacodylate buffer (Structure Probe Incorporated, West Chester, PA), 50% glutaraldehyde, aqueous 16% paraformaldehyde (Structure Probe Incorporated, West Chester, PA) and cation stock solution consisting of 0.3% CaCl_2_ and 0.5% MgSO_4_ in 100 ml of DI water. Samples were then washed with DI water five times for 10 minutes each. Following the washes, samples were dehydrated with 50, 70 and 95% ethanol (EtOH) 15 min each and dehydrated with 100% EtOH, twice for 15 min each. Consequently, samples were dried with a critical point dryer, sputtercoated with platinum/palladium and mounted onto S stubs (Structure Probe Incorporated, West Chester, PA) with carbon tape and paint (Structure Probe Incorporated, West Chester, PA). Surface images were taken with a Zeiss Supra40 Scanning Electron Microscope (Zeiss, Oberkochen, Germany).

Analysis of the SEM images was carried out using ImageJ^®^ software (Bethesda, MD). The hydrogel porosity was measured by taking a 10k × magnification image of each hydrogel sample and adjusting the threshold of all the images from 0 to 145 to isolate the fibers. The area of the image not occupied by fibers was considered the porous region. This percentage was considered as the porosity of the hydrogel. The fiber orientation was quantified using the directionality plugin in ImageJ^®^ (Bethesda, MD) on representative figures for each sample to generate a histogram of fibers’ directions in angles. For the fiber width, 20 fiber width measurements were taken from 50k × magnification images (*n* = 9) and displayed as a histogram.

### 2.6. Rheology

Rheology was performed on polymerized acellular hydrogels to determine whether the addition of KTN affected the storage modulus (stiffness). A strain sweep was first conducted with a 25 mm ETC steel plate (TA Instruments Ares 2000ex rheometer, Newcastle, DE) on all hydrogels to determine the strain rate in the linear viscoelastic (LVE) region. One percent strain rate was chosen for the frequency sweep from 0.1 to 15 Hz to determine each hydrogel’s storage modulus. *G*′ (storage modulus) and *G*′′ (loss modulus) were plotted from 0.1 to 15 Hz. Storage modulus values for each hydrogel were recorded at 1 Hz in the LVE region, as most of the literature concludes that 1 Hz produces a linear response in collagen hydrogels [22].

### 2.7. Differential scanning calorimetry

Acellular gels were prepared for DSC to determine whether the addition of KTN increased the *T*_m_ and, in effect, the hydrogel’s thermal stability. A hydrogel section weighing between 5 and 10 mg was cut, weighed and recorded, and placed into separate tzero pans (TA Instruments, Newcastle, DE). Pans were closed with tzero hermetic lids (TA Instruments, Newcastle, DE) and placed on the DSC’s reference sensor (TA Instruments DSC 2000, Newcastle, DE). Samples were heated from 30 to 80 °C at a rate of 5 °C/min.

### 2.8. Temperature stability

To determine whether addition of KTN increased the thermal stability of the hydrogel and enabled maintenance of their shape when exposed to hyperthermic temperature, hydrogels were weighed after thermal degradation. Single cut 24-well plate size wells were weighed and recorded, and cellular hydrogels were made in every well and polymerized as discussed previously and stored in PBS at 37 °C at 5% CO_2_. PBS was removed, and polymerized acellular hydrogels were weighed and placed in a sterile incubator at 5% CO_2_ at 47 °C for 20, 30 and 60 min. 47 °C was chosen, as hyperthermia treatment is considered a rise in temperature ranging from 40 to 47 °C, so the highest temperature in that range was selected [29,71]. After each time point, parts of the hydrogel that degraded/melted were removed by blotting carefully with a fiber-optic cleaning wipe, and the well with the remaining solid hydrogel material was weighed. The following equation was used to calculate the normalized weight:

$$\text{Normalized weight \%}=\frac{M_{1}-M_{2}}{M_{1}}\times 100\%$$

where *M*_1_ and *M*_2_ are the weight before and after incubation, respectively.

### 2.9. Cell viability

The viability of cells seeded in hydrogels was quantified to determine whether the addition of KTN had any cytotoxic effect. The CellTiter-Blue^®^ cell viability assay (Promega, Madison, WI) was used to measure the cell viability of each sample at days 1, 3, 5 and 7 post-seeding. The complete media in each sample was replaced with 100 μl of fresh complete media and 20 μl of CellTiter-Blue^®^ reagent. Samples were incubated for 2 h for NHDF and 1 h for MDA-MB-231 breast cancer cells. The supernatant for each sample was then transferred to clean separate wells, and fluorescence was measured with a plate reader at an emission of 560 nm and excitation at 590 nm. Background fluorescence intensity (blank well with media and CellTiter-Blue^®^ reagent) was subtracted from sample readings.

### 2.10. Cell metabolic activity

The CellTiter-Glo^®^ Luminescent cell viability assay (Promega, Madison, WI) was used to quantify the level of ATP present in each sample, an indication of metabolically active cells. Although the CellTiter Blue assay discussed in the previous section can provide information on the overall viability of the cells seeded in each hydrogel, they may be more metabolically active in one hydrogel over another. Therefore, cell metabolic activity was also measured at the same time points. On days 1, 3, 5 and 7, samples were first incubated in 2.5 mg/ml collagenase (Roche Diagnostics, Basel, Switzerland) solution of equal volume to the hydrogel for 1 h to digest the hydrogels and isolate the cells. The resulting solution from each sample was centrifuged at 200×*g* for 3 min and the cell pellets for each sample were then isolated individually and incubated in 100 μl of CellTiter-Glo solution for 10 min. Luminescence intensity was recorded with a plate reader. Background luminescence was subtracted from sample readings.

### 2.11. Actin and DAPI staining and aspect ratio quantification

To observe cell morphology, cells in hydrogels were stained for actin and DAPI to count the number of cells over time. Cellular hydrogels were fixed with 3.7% paraformaldehyde (MP Biomedicals, Santa Ana, CA) at days 1, 3, 5 and 7 for 15 min at room temperature and consequently washed with PBS twice for 5 min each. Samples were then permeabilized with 0.1% Triton X-100 (Sigma-Aldrich, St. Louis, MO) dissolved in PBS and washed with PBS twice for 5 min each. Samples were blocked with 5% BSA (Fisher Scientific, Hampton, NH) dissolved in PBS for 30 min and consequently stained with Alexa-Fluor^™^ 488 phalloidin (Invitrogen, Carlsbad, CA) at a dilution of 1:100 for 20 min to observe cell morphology. Samples were once again washed twice with PBS for 5 min and stained with one drop of DAPI Fluoromount-G (Southern Biotech, Birmingham, AL) for 5 min. One hundred microliters of PBS was added to each sample, which was then imaged with a confocal microscope (Leica Microsystems, Wetzlar, Germany) with a 2 × 2 tile-scan at 10× magnification over a thickness of 600 μm and an excitation/emission of 495/518 nm to image actin, and 358/461 nm to image nuclei (DAPI). Post-processing was done by adjusting the gain and contrast of the stacked 3D image volume and rotating the image to observe from the top of the hydrogel in the Leica LASX software (Wetzlar, Germany). The AR was quantified by measuring the width and length of nine random cells from each image (12 per sample and per time point) and dividing the length by the width. Measurements were made employing ImageJ^®^ software (Bethesda, MD) by calibrating the length of the scale bar to 200 μm and manually measuring the width and length of each cell.

### 2.12. Live cell number quantification

Samples at days 1, 3, 5 and 7 were stained with calcein-AM to determine the number of live cells. Cells were counted to quantify cell growth over time to determine whether the addition of KTN in the hydrogels inhibited or promoted cell growth. At each time point, cell media was replaced with 100 μl of 4 μM calcein-AM solution in PBS and incubated for 20 min at 37 °C. Subsequently, the solution was aspirated and rinsed with PBS for 2 min. Samples were then replaced with fresh PBS and immediately imaged with a confocal microscope. Each sample was imaged at excitation/emission of 490/515 nm with a z-projection of 150 μm. Images were processed from the top view, and the total live cell number was quantified by counting the number of calcein-AM stained cells using ImageJ^®^ software (Bethesda, MD). This count was then converted to the cell number/ml of the hydrogel. For each sample, 12 images were analyzed per time point (days 1, 3, 5 and 7) and plotted over time.

### 2.13. Statistical analysis

Data for each test were analyzed for significance using one-way ANOVA. Comparisons were only made between 100% collagen (control) and each C/KTN hydrogel to determine significance. Significance was also compared between different time points for cellular experiments within all hydrogel samples separately (*p* < .05 and *p* < .01). Results are represented as either weighted or overall average with standard deviations of three or more repeated experiments. When making comparisons, C/KTN hydrogels will refer to all 50/50, 30/70 and 20/80 C/KTN hydrogels. Three milligrams per milliliter or 7 mg/ml hydrogels refers to all 100% collagen 50/50 C/KTN, 30/70 C/KTN and 20/80 C/KTN hydrogels of that specific collagen concentration (3 or 7 mg/ml).

## 3. Results

### 3.1. Hydrogel structure and ECM analysis

**Figure 1.**
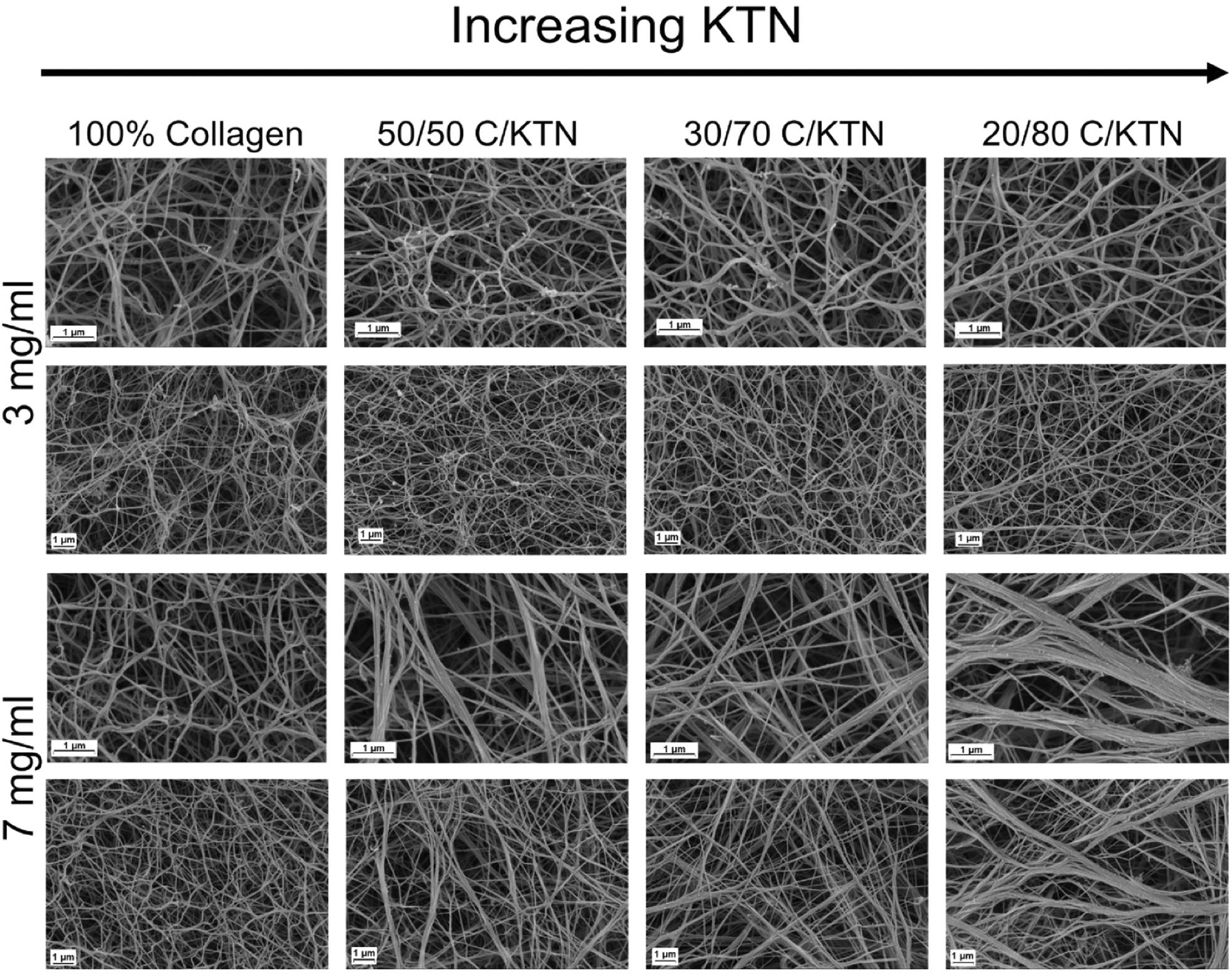
ECM structure of hydrogels imaged with SEM. Representative SEM images of 3 and 7 mg/ml collagen hydrogels with increasing KTN content at ×50,000 and ×25,000 magnification. Second and 4th rows represent the zoomed out images of the first and third rows, respectively. Three milligrams per milliliter hydrogels appeared to exhibit minimal difference in fiber structure with increased KTN concentration; on the other hand, fiber structure of 7 mg/ml hydrogels changed with increase in KTN concentration as porosity decreased and fiber bundling increased.

Cells grown in a 3D environment can behave differently compared to 2D due to the fiber network, allowing them to make adhesive contacts with surrounding cells and the ECM [72]. In particular, fibroblasts become more spindle-shaped in an *in vitro* 3D microenvironments compare to *in vivo* [73,74]. This is owed to the physical restriction of the ECM fibrils, including collagen fibrils present in native ECM [72,73]. In contrast, invasive cancer cells, including MDA-MB-231 breast cancer cells, demonstrate stellate morphology and projections in 3D culture and *in vivo* [75]; therefore, it is important to study the ECM structure of hydrogels by using SEM (Figure 1). As expected, significantly higher porosity was measured for 3 mg/ml compared to 7 mg/ml hydrogels (Figure S1A) for all conditions (*p* < .05). For 3 mg/ml hydrogels, 100% collagen hydrogels had significantly higher porosity (70.4 ± 3.4%) compared to 20/80 C/KTN hydrogels (67.1 ± 2.2%,). Seven milligrams per milliliter 100% collagen hydrogels (66.8 ± 1.2%) had significantly higher porosity than 50/50 (64.0 ± 2.3%), 30/70 (64.3 ± 1.5%) and 20/80 (62.4 ± 2.1%) C/KTN hydrogels with *p* < .01. Further, we analyzed the fiber width of samples and found an increase in fiber width with increasing KTN concentration for 7 mg/ml hydrogels. As demonstrated by the histogram in Figure S1B, 7 mg/ml C/KTN hydrogel samples possessed a higher density of fibers with increased fiber widths of 151–420+ nm compared to 100% collagen hydrogels in which most were in the range of 61–80 nm. On the other hand, fibers of 3 mg/ml C/KTN hydrogels showed no difference when compared to 100% collagen hydrogels, demonstrating KTN did not increase bundling of fibers in 3 mg/ml hydrogels. Directionality of fibers was also measured by ImageJ and plotted as percentage of fibers for the specific angle as shown in Figure S1C. As demonstrated, a rise in KTN in 3 and 7 mg/ml hydrogels increased the percentage of specific angles in one direction as shown by peaks in the histogram. This is interpreted as fibers that were aligned rather than randomly distributed, which is shown in both 3 and 7 mg/ml 100% collagen hydrogels with a linear histogram. This single-direction organization of fibers was more prominent in 7 mg/ml hydrogels, showing higher peaks compared to 3 mg/ml samples. These results reflected the observations made by SEM images shown in Figure 1.

### 3.2. Mechanical properties of hydrogels

**Figure 2.**
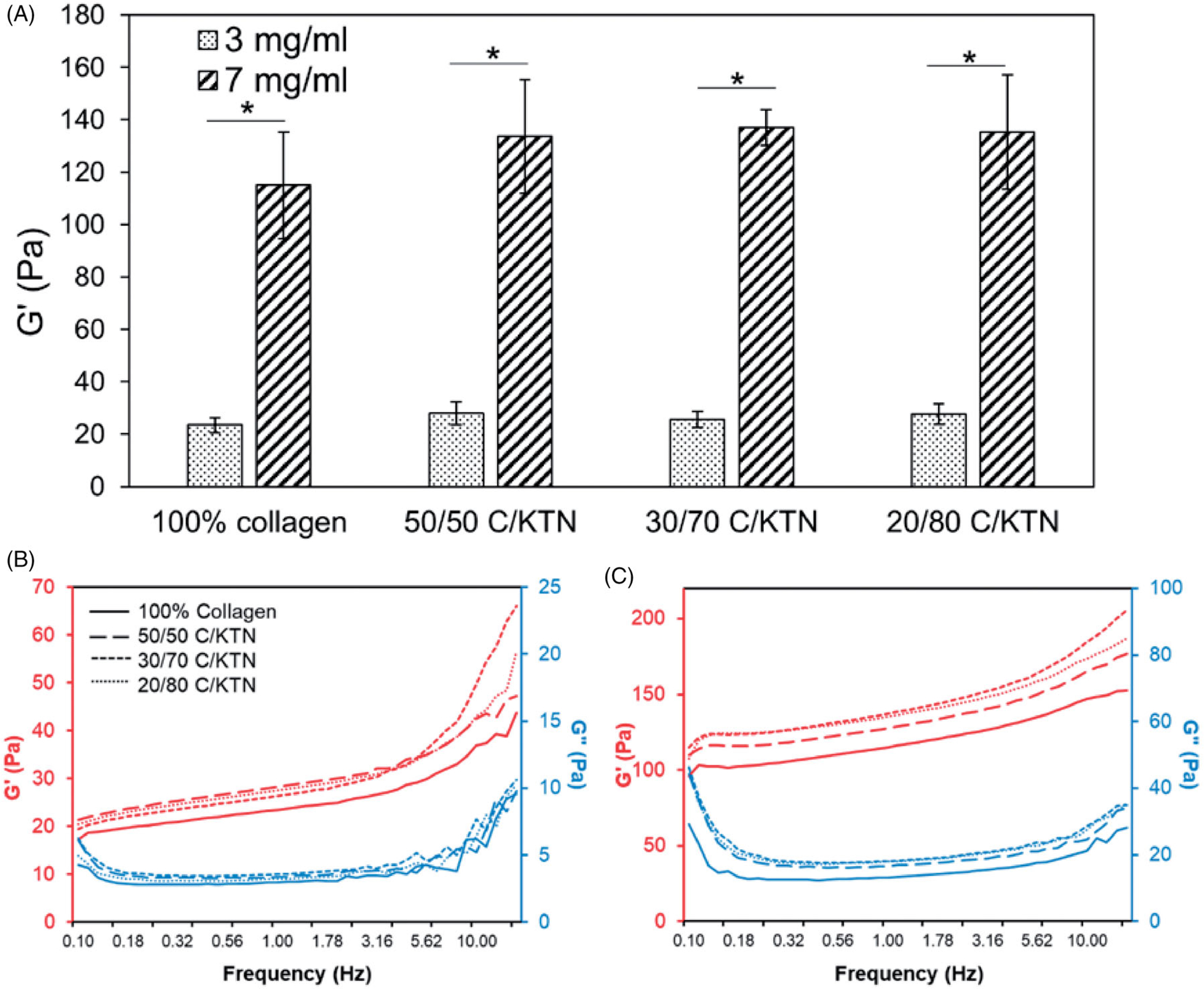
Stiffness of 100% collagen and C/KTN hydrogels. Storage modulus measured by an oscillation sweep from 0.1 to 15 Hz at 1% strain and analyzed at 1 Hz (three independent experiments, *n* = 4–5 for each experiment). The storage modulus increased significantly with greater collagen concentration from 3 to 7 mg/ml. Although higher KTN concentration increased the storage modulus, the values were not significantly different from the storage modulus of 100% collagen hydrogels. One-way ANOVA performed, results are weighted average ± SD, *p<.05. Average storage and loss modulus of 3 (B) and 7 mg/ml (C) hydrogels were plotted versus frequency at 1% strain (*n* = 9). Results show that increase in KTN concentration led to an upward shift in the plot, representing higher storage modulus.

Rheological testing of hydrogels was conducted to determine the hydrogel storage modulus and the effect of collagen and keratin concentration on this metric. As shown in Figure 2(A), 3 mg/ml hydrogels had a storage modulus between 23.5 and 27.8 Pa, which was significantly lower (*p* < .01) than 7 mg/ml hydrogels (115.0–135.2 Pa). Significant differences were not observed between 100% collagen and C/KTN hydrogels. Overall, increasing collagen concentration has a more significant impact than KTN concentration on sample stiffness. *G*′ and *G*′′ plots demonstrated in Figure 2 also showed that *G*′′ is lower than *G*′ for the entire frequency range (0.1–15 Hz).

### 3.3. Melting temperature (*T*_m_)

**Figure 3.**
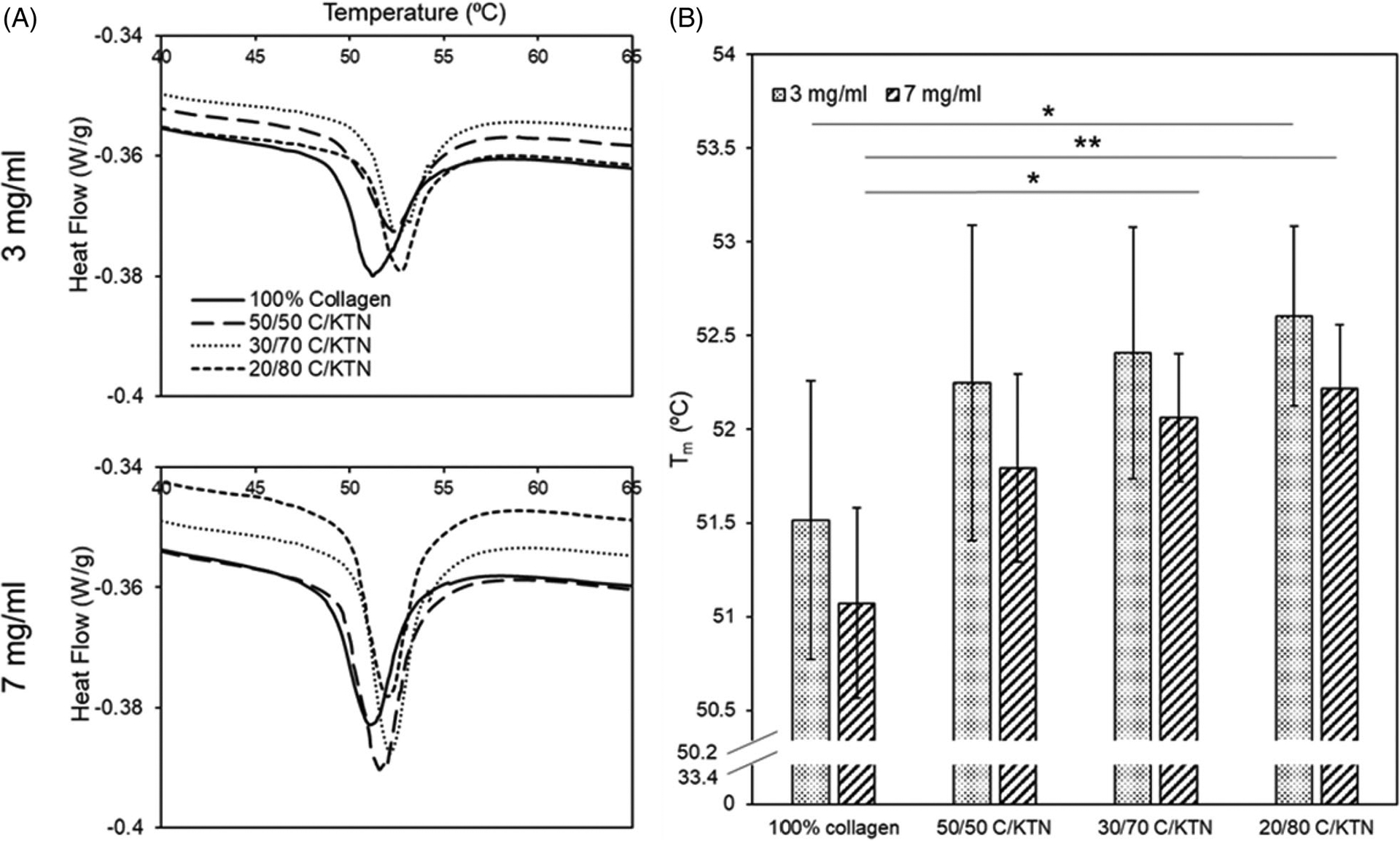
DSC denaturing temperature. Average DSC peaks of hydrogel samples for both 3 and 7 mg/ml hydrogels (*n =* 15) (A). DSC peaks were plotted as a bar graph to display the denaturing temperature of each hydrogel (five independent experiments with *n* = 3 for each experiment) (B). In general, increase in *T*_m_ (peaks) corresponded with increase in KTN concentration. Results are weighted average ± SD, One-way ANOVA was performed with *n* = 9. *p<.05, **p<.01.

DSC was used to determine whether the addition of KTN increased the *T*_m_ of the hydrogels. The *T*_m_ peaks of each hydrogel increased as KTN concentration rose, as demonstrated in Figure 3. Significant increases in *T*_m_ were only observed between 3 mg/ml 100% collagen and 20/80 C/KTN hydrogels (*p* < .05), 7 mg/ml 100% collagen and 30/70 C/KTN (*p* < .05) and 7 mg/ml 100% collagen and 20/80 C/KTN hydrogels (*p* < .01). However, a trend in increasing *T*_m_ was observed with an increase in KTN content (Figure 3(A,B)).

### 3.4. Thermal degradation and hydrogel stability

**Figure 4.**
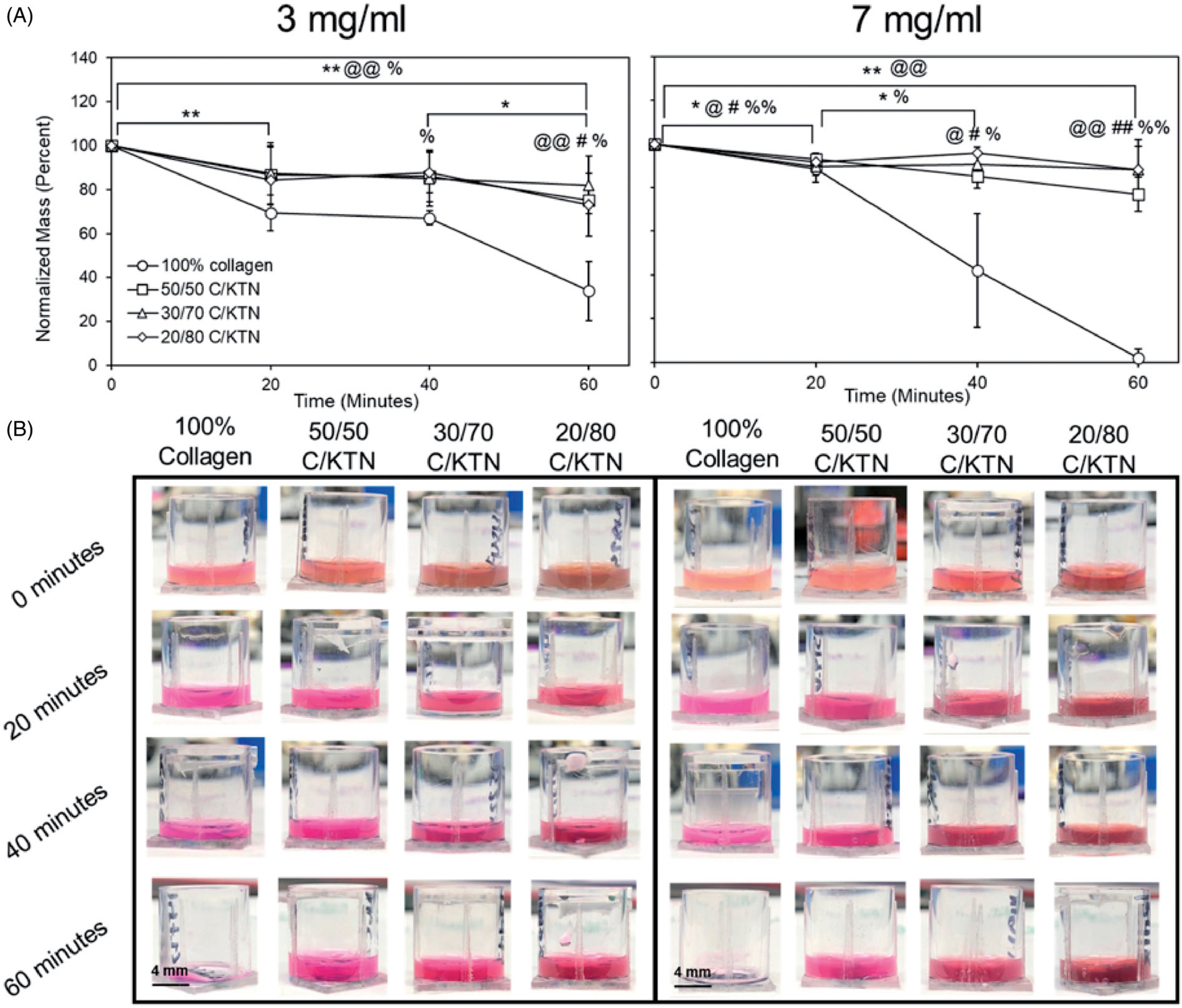
Hydrogel thermal stability at 47 °C. Normalized mass percentage was calculated based on normalizing the mass measured every 20 min up to 1 h to the original mass (A). Representative images of 100% collagen and C/KTN hydrogels at varying time points (B). After 60 min, all C/KTN hydrogels remained intact whereas 100% collagen hydrogels were almost or completely melted. Results are average ± SD, significance is demonstrated as: *_100%_ collagen, ^@^50/50, ^#^30/70, ^%^20/80; *p<.05, **p<.01. Notations refer to comparisons made between time points of each sample (if above significance bars) and between samples at each timepoint (if above a certain timepoint). Three independent experiments were conducted with *n* = 3–8 for each experiment. Scale bar = 4 mm.

Water loss due to hyperthermia was quantified by measuring the weight of the hydrogels post-incubation at 47 °C for up to 60 min. Three milligrams per milliliter C/KTN hydrogels showed significantly higher normalized mass than 100% collagen after 60 min of incubation. After 20 min, 3 mg/ml collagen hydrogels were the only samples that had a significant loss (*p* < .01). From 40 to 60 min, there was a significant decrease in normalized mass with only 100% collagen hydrogels (33.8 ± 13.4%, *p* < .05). Overall, significant decrease (0–60 min) in normalized mass was observed with 100% collagen (*p* < .01), 5050 C/KTN (*p* < .01) and 20/80 C/KTN hydrogels (*p* < .05). After 60 min of incubation, 50/50 (2.2-fold, *p* < .01), 30/70 (2.4-fold, *p* < .05) and 20/80 (2.2-fold, *p* < .05) C/KTN hydrogels had significantly higher normalized mass than 100% collagen hydrogels. As demonstrated by Figure 4(B), there was little to no hydrogel remaining at 60 min for 3 mg/ml hydrogels with an average of 30%, whereas all C/KTN hydrogels maintained their shape with the presence of a solid hydrogel.

Similar results were also observed with 7 mg/ml hydrogels, as shown in Figure 4(A). After 40 min, all 7 mg/ml C/KTN hydrogels had about twofold higher (*p* < .05) normalized mass than 100% collagen hydrogels. Overall, there was a significant decrease in normalized mass from the original mass with 100% collagen (*p* < .01) and 50/50 C/KTN (*p* < .01) hydrogels. In addition, the normalized mass of C/KTN hydrogels had about 40-fold higher (*p* < .01) normalized mass compared to 100% collagen hydrogels after 60 min of incubation. In contrast to 7 mg/ml collagen hydrogels, all C/KTN hydrogels maintained their shape with the presence of a solid hydrogel after 60 min of incubation (Figure 4(B)).

### 3.5. Cell viability in hydrogels

**Figure 5.**
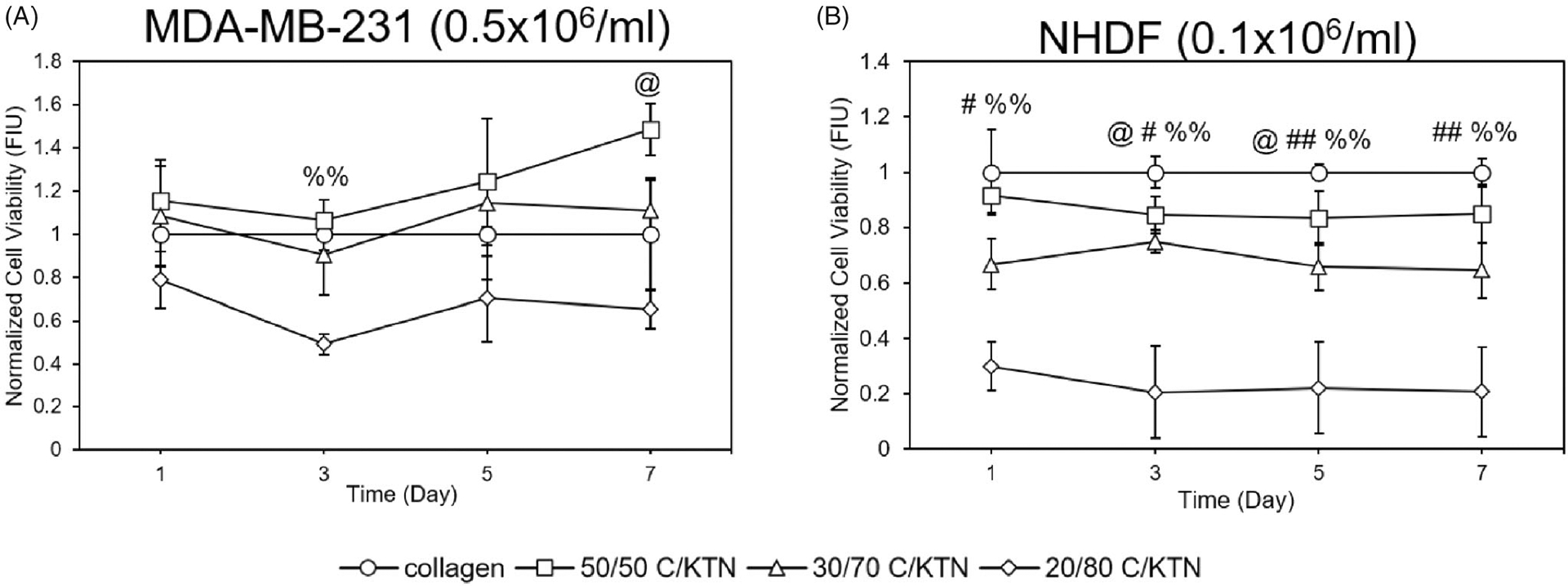
Cell viability of MDA-MB-231 breast cancer cells and NHDFs seeded in various hydrogels. The normalized cell viability (to 100% collagen hydrogels) was measured through CellTiter Blue viability assay at 1, 3, 5 and 7 days post cell seeding for MDA-MB-231 breast cancer cells seeded at 0.5 million/ml (A) and NHDFs seeded 0.1 million/ml (B) and displayed as fluorescence intensity unit (FIU). MDA-MB-231 seeded 50/50 C/KTN hydrogels showed significantly higher viability than 100% collagen hydrogels at days 5 and 7, but lower viability for 20/80 C/KTN hydrogels at days 1 and 3 (A). NHDFs seeded in C/KTN hydrogels had significantly lower viability when compared to 100% collagen hydrogels at all time points (B). Results are demonstrated as weighted average ± SD with three independent experiments with *n* = 4 for each experiment. One-way ANOVA performed to compare C/KTN hydrogels with 100% collagen hydrogel with significance demonstrated as: ^@^50/50, ^#^30/70, ^%^20/80; single indicator signifies *p* < 0.05 and double indicator signifies *p* < 0.01.

The viability of cells in C/KTN hydrogels was quantified to determine whether the addition of KTN had any effect on cell viability. To directly compare results to 100% collagen, viability results of cells seeded in C/KTN hydrogels were normalized to that of cells seeded in 100% collagen hydrogels, as shown in Figure 5. Overall, from days 3 to 7, normalized viability of breast cancer cells seeded in 20/80 C/KTN hydrogels was lower than that of cells seeded in 100% collagen, with lower viability at day 3 (*p* < .01) as shown in Figure 5(A). On day 7, cell viability of breast cancer cells seeded in 50/50 C/KTN hydrogels was higher (*p* < .05) than that of cells seeded in 100% collagen hydrogels.

As shown in Figure S2A, the viability of NHDFs seeded in 3 mg/ml C/KTN at 0.5 × 10^6^/ml NHDFs/ml had no significant changes compared to 100% collagen hydrogels, possibly due to initial high seeding density. However, when seeded at 0.1 × 10^6^/ml, the viability of NHDFs seeded in C/KTN hydrogels was lower at each time point, with the lowest normalized viability observed in 20/80 C/KTN hydrogels (Figure 5(B)).

### 3.6. Cell metabolic activity in hydrogels

**Figure 6.**
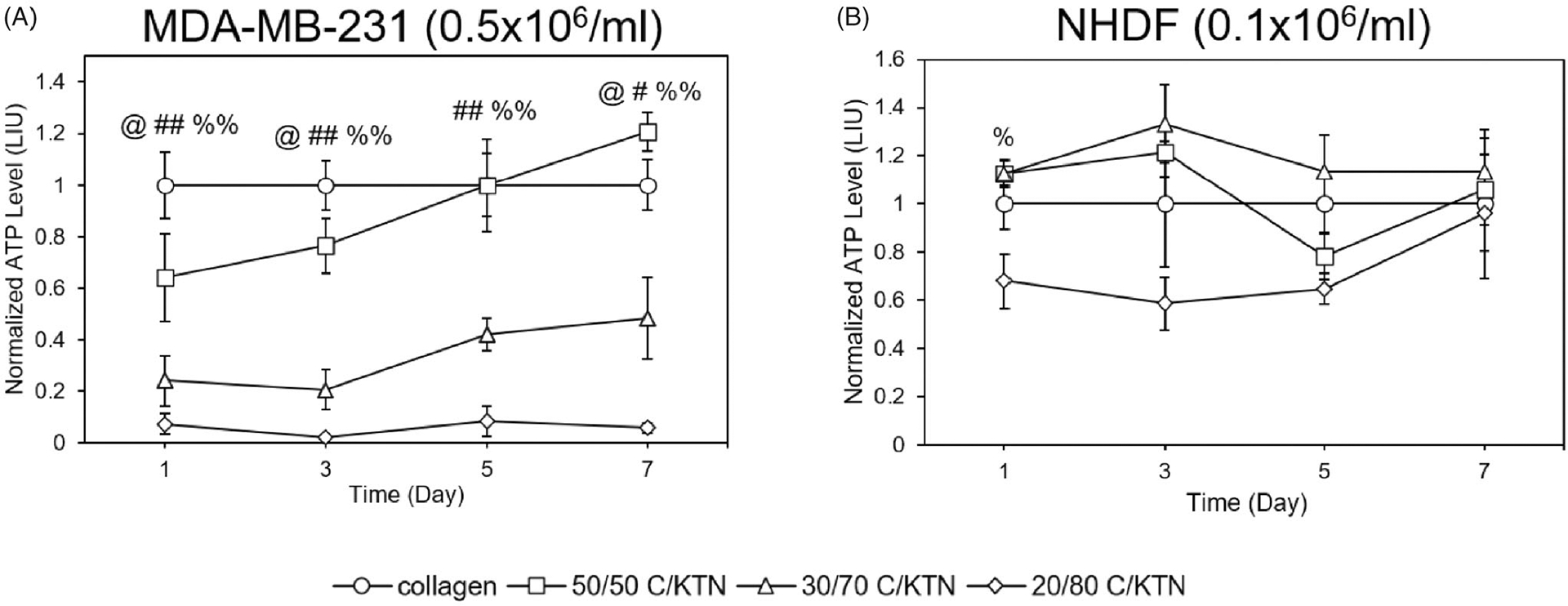
Cell ATP level of MDA-MB-231 breast cancer cells and NHDFs seeded in various hydrogels. Normalized cell ATP activity (to 100% collagen hydrogels) was measured through CellTiter Glo cell metabolic assay at 1, 3, 5 and 7 days post cell seeding for MDA-MB-231 breast cancer cells seeded at 0.5 million/ml (A) and NHDFs seeded at 0.1 million/ml (B) and displayed as luminescence intensity unit (LIU). MDA-MB-231 seeded in 50/50 C/KTN hydrogels showed significantly higher metabolic activity than 100% collagen hydrogels at day 7, but lower viability at day 3 (A). Cells seeded in 30/70 and 20/80 C/KTN hydrogels had significantly lower metabolic activity than those seeded in 100% collagen at all time points (A). NHDFs seeded at 0.1 million cells/ml in C/KTN hydrogels had comparable metabolic activity to 100% collagen hydrogels (B). Results are demonstrated as weighted average ± SD with three independent experiments with *n =* 4 for each experiment. One-way ANOVA performed to compare C/KTN hydrogels with 100% collagen hydrogel with significance demonstrated as: ^@^50/50, ^#^30/70, ^%^20/80; single indicator signifies *p* < 0.05 and double indicator signifies *p* < 0.01.

ATP levels of MDA-MB-231 breast cancer cells and NHDFs in C/KTN hydrogels were quantified through the CellTiter Glo luminescent assay to determine whether the addition of KTN had any effect on cell metabolism. At each time point, MDA-MB-231 breast cancer cells seeded in 7 mg/ml 30/70 and 20/80 C/KTN hydrogels had lower metabolic activity compared to that of collagen (*p* < .01, *p* < .05 for 30/70 day 7, Figure 6(A)). However, cells seeded in 50/50 C/KTN hydrogels had lower (*p* < .05) normalized metabolic activity at days 1 and 3, metabolic activity was higher (1.2-fold, *p* < .05) than that of 100% collagen at day 7. Similarly, a significant increase in cell viability was only observed with 50/50 C/KTN hydrogels on day 7 (Figure 5(A)).

Similar to cell viability, the metabolic activity of NHDFs seeded in C/KTN hydrogels at 0.5 × 10^6^/ml was not different from 100% collagen hydrogels, with significant decreases in normalized metabolic activity only observed with 20/80 C/KTN hydrogels at day 1 and 7 (*p* < .05) (Figure S2B). NHDFs seeded at 0.1 × 10^6^/ml displayed metabolic activity that differed from cell viability results (Figure 6(B)). Whereas cells seeded in 30/70 and 20/80 C/KTN hydrogels had lower normalized viability than 100% collagen hydrogels at all time points, lower normalized metabolic activity was only observed with 20/80 C/KTN hydrogels at day 1 (*p* < .05) with no significant difference observed between C/KTN hydrogels and 100% collagen at all other time points (Figure 6(B)).

### 3.7. Cell morphology and spreading

**Figure 7.**
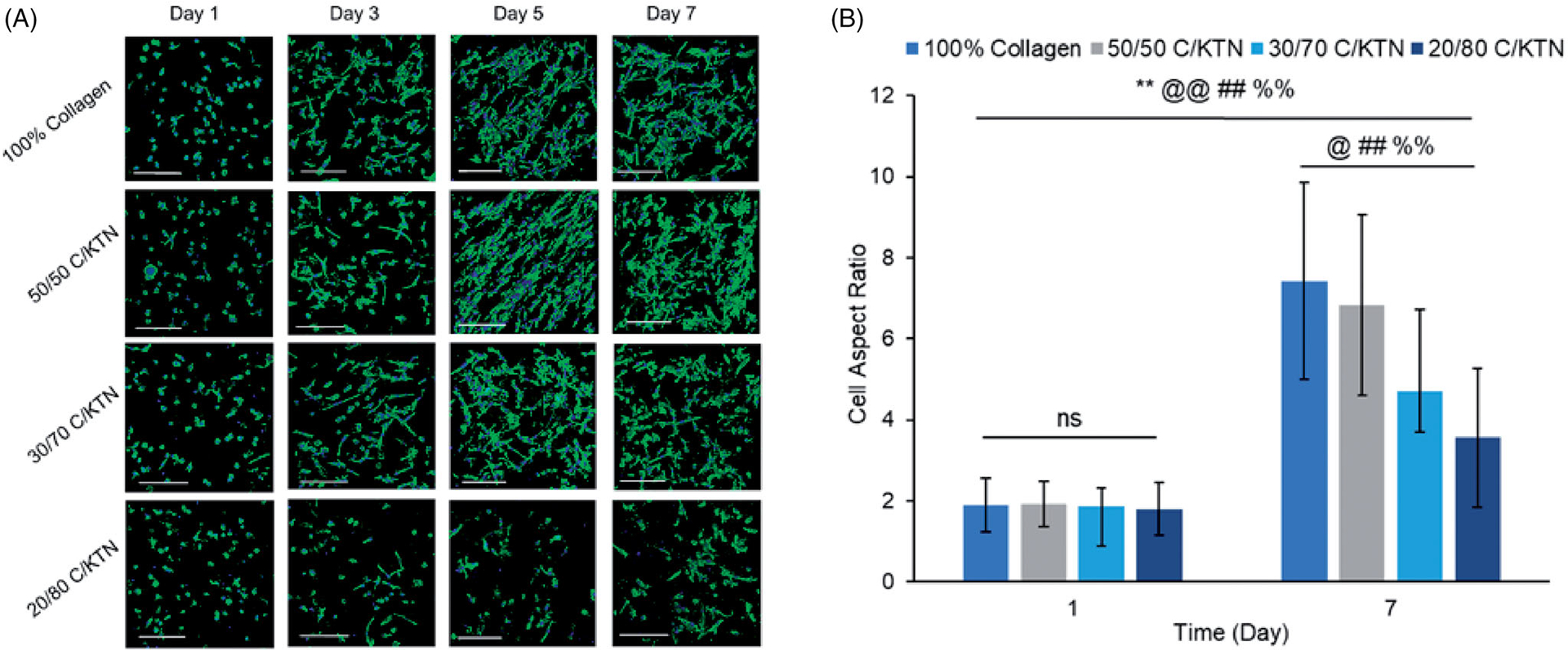
Cell morphology of aspect ratio of MDA-MB-231 breast cancer cells seeded at 0.5 million cells/ml. Confocal images of MDA-MB-231 breast cancer cells stained with phalloidin 488 and DAPI at 1, 3, 5 and 7 days post cell seeding (A) with measured aspect ratio at days 1 and 7 (B). Cells in all samples appeared more elongated and proliferated by day 7 with significantly higher aspect ratio from day 1 to 7 (*p*<.01) and higher aspect ratio observed in collagen (*p*<.01) compared to each C/KTN hydrogel. Samples were imaged at a total depth of 600 μm. Scale bar = 200 μm. Results are demonstrated as weighted average ± SD with three independent experiments with *n* = 4 for each experiment. One-way ANOVA performed to compare between time points of each sample with notations (*=100% collagen, @=50/50 C/KTN, #=30/70 C/KTN, %=20/80 C/KTN) above significance bars. Comparisons also made between 100% collagen hydrogels and each C/KTN hydrogel at each time point. Single notation indicates *p* < 0.05 and double notation indicates *p* < 0.01.

Cell morphology over time was monitored by confocal imaging of MDA-MB-231 breast cancer cells and NHDFs stained for filamentous actin (F-actin) over seven days. The corresponding AR was also measured and plotted over time. Morphology of MDA-MB-231 breast cancer cells imaged by fluorescent confocal microscopy showed that the presence of KTN did not inhibit cell spreading by day 7 (Figure 7(A)) with no significant difference observed between 100% collagen and C/KTN hydrogels (Figure 7(B)). Initially, however, 20/80 C/KTN hydrogels contained cells that were still spherical at day 3 with a lower AR, whereas cells seeded in the pure collagen, 50/50 C/KTN and 30/70 C/KTN hydrogels were already starting to elongate at day 1. By day 7, there was a noticeable cell number increase for all hydrogels observed by previous studies showing active growth [75,76] except for 20/80 C/KTN hydrogels. These images correlate with the cell viability (Figure 5(A)) and metabolic activity (Figure 6(A)) results in which 20/80 C/KTN hydrogels had the lowest normalized values.

**Figure 8.**
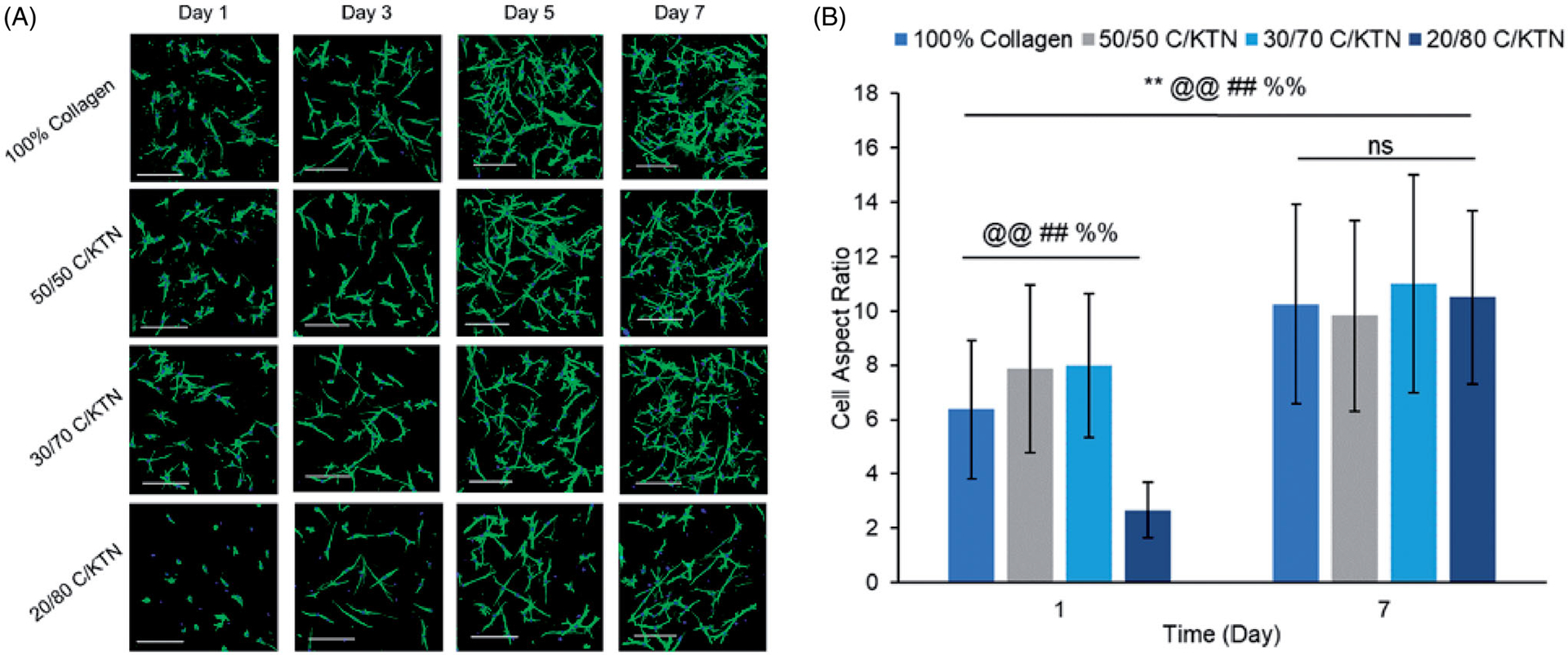
Cell morphology of NHDFs seeded at 0.1 × 10^6^ cells/ml. Confocal images of NHDFs stained with phalloidin 488 and DAPI at 1, 3, 5 and 7 (A) and measured aspect ratio at days 1 and 7 (B). Cells in all samples appeared more elongated and proliferated by day 7 with significantly higher aspect ratio from day 1 to 7 (*p*<.01) and higher aspect ratio observed in 50/50 and 30/70 C/KTN hydrogels when compared to pure collagen at day 1 (*p*<.01). 20/80 C/KTN hydrogels appeared more spherical and had significantly lower aspect ratio when compared to 100% collagen at day 1 (*p*<.01), but had similar aspect ratio at day 7. Samples were imaged at a total depth of 600 μm. Scale bar = 200 μm. Results are demonstrated as weighted average ± SD with three independent experiments with *n =* 4 for each experiment. One-way ANOVA performed to compare between time points of each sample with notations (*=100% collagen, @= 50/50 C/KTN, #=30/70 C/KTN, %=20/80 C/KTN) above significance bars. Comparisons also made between 100% collagen hydrogels and each C/KTN hydrogel at each time point. Single notation indicates *p* < 0.05 and double notation indicates *p* < 0.01.

NHDFs seeded at 0.5 × 10^6^/ml in C/KTN hydrogels had a similar appearance to NHDFs seeded in 100% collagen hydrogels (Figure S2C). These results correlate with the normalized cell viability (Figure S2A) and metabolic activity (Figure S2B), in which little to no significance was observed between 100% collagen and C/KTN hydrogels. When NHDFs were seeded at 0.1 million cells/ml, cell growth and proliferation were visualized over seven days in all the hydrogels (Figure 8(A)) with an increased AR from day 1 to 7 for all samples (Figure 8(B)). However, on day 1, cells seeded in 20/80 C/KTN hydrogels appeared more spherical than the cells seeded in other samples with a significantly lower AR.

### 3.8. Live cell number and growth over time

**Figure 9.**
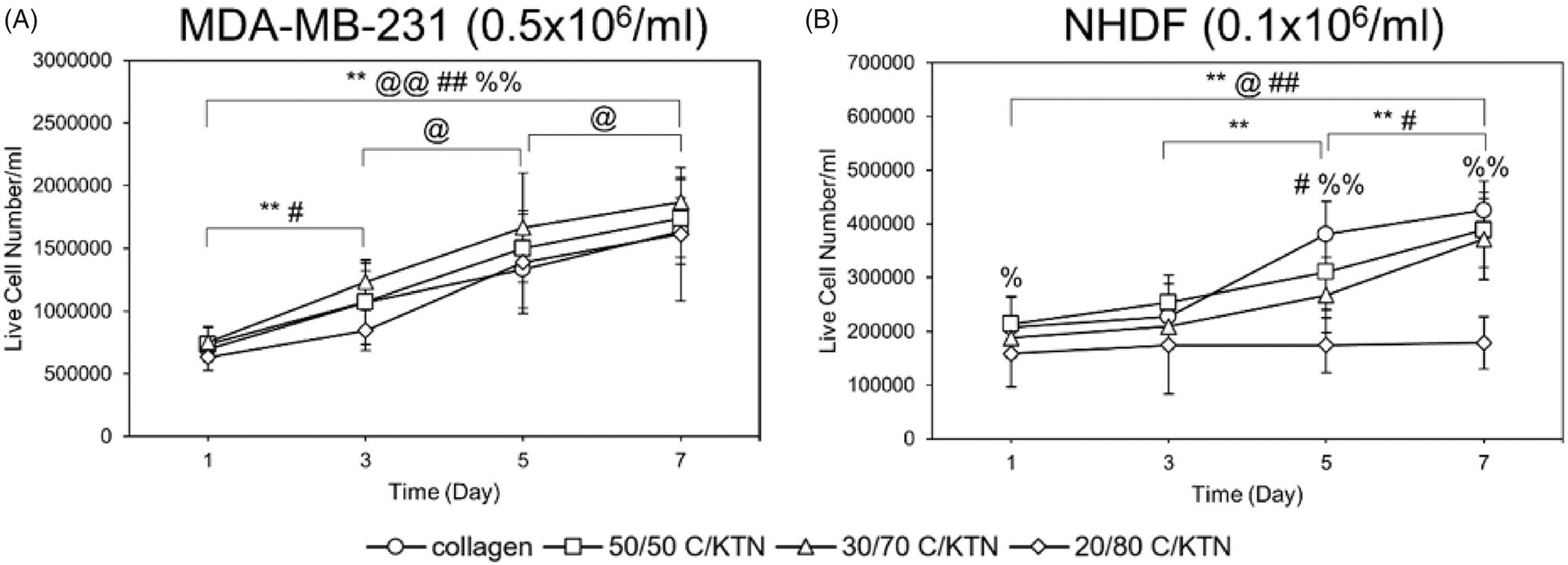
Live cell number of calcein AM-stained cells. MDA-MB-231 breast cancer cells seeded at 0.5 × 10^6^/ml (A) and NHDFs seeded at 0.1 × 10^6^/ml (B) were stained with calcein-AM at days 1, 3, 5, 7 and imaged with a confocal microscope for a total thickness of 150 μm. Z projection images were used to count the total number of live cells using ImageJ^®^ software (Bethesda, MD). Overall, cell proliferation was observed except with NHDFs when seeded with 3 mg/ml 20/80 C/KTN hydrogels. One-way ANOVA was performed to compare between time points of each sample with notations (*=100% collagen, @= 50/50 C/KTN, #=30/70 C/KTN, %=20/80 C/KTN) above significance bars. Comparisons also made between 100% collagen hydrogels and each C/KTN hydrogel at each time point. Single notation indicates *p* < 0.05 and double notation indicates *p* < 0.01.

Images of calcein-AM staining captured by confocal microscopy were used to count the number of live cells employing ImageJ^®^ software (Bethesda, MD). As there was no observable cell growth with NHDFs when seeded at 0.5 × 10^6^ cells/ml, only hydrogels seeded at 0.1 × 10^6^ NHDFs/ml were evaluated. As shown by Figure 9(A), significant cell growth (*p* < .01) of MDA-MB-231 breast cancer cells was observed from day 1 to 7 in all hydrogel formulations. In addition, there was no significant difference in cell growth between each C/KTN hydrogel formulation and 100% collagen at each time point, indicating that the cell growth in C/KTN hydrogels was similar to that of cell growth to the control group of 100% collagen. These results are mostly similar to cell viability results, in which there was no significant difference between samples except for 20/80 C/KTN (day 3) and 50/50 C/KTN (day7) (Figure 5(A)).

Only NHDFs seeded within 100% collagen, 50/50 C/KTN and 30/70 C/KTN hydrogels showed significant cell growth from day 1 to 7 (Figure 9(B)). NHDFs seeded within 20/80 C/KTN hydrogels had no significant growth over seven days, possibly due to restriction of the high protein concentration. Similarly, the normalized cell viability of NHDFs seeded in C/KTN hydrogels had significantly lower cell viability than 100% collagen (Figure 5(B)). Although we acknowledge the cell viability of NHDFs seeded in 50/50 and 30/70 C/KTN hydrogels is significantly lower than 100% collagen for most time points, it is 3.25- to 4.5-fold higher than the viability of 20/80 C/KTN hydrogels. We also acknowledge they are as metabolically active as the cells seeded in 100% collagen (Figure 6(B)), with observable cell growth and spreading (Figure 8).

## 4. Discussion

3D *in vitro* culture models employ numerous types of hydrogels, including natural and synthetic. Natural biomaterials are attractive because they are present *in vivo* and include endogenous factors for optimal cell growth [77]. Collagen proteins, which make up 25% of the whole-body protein content *in vivo*, have been widely used for tissue and biomimetic engineering applications, and therefore was used as a comparative baseline for C/KTN blend hydrogels in this study [78,79]. To increase collagen’s thermal properties to be employed in thermal stress studies and better mimic the denaturing point of human tissue, KTN was added at increasing concentrations. We demonstrated that the addition of KTN increased the thermal stability after exposure to hyperthermic temperatures with 50/50 C/KTN supporting similar or higher cell growth than pure collagen hydrogels.

C/KTN hydrogels were characterized for structural, mechanical and thermal properties compared to properties of pure 3 and 7 mg/ml collagen hydrogels to mimic mechanical properties of healthy and cancerous tissues, respectively. An increase in collagen concentration decreased the porosity due to increased fiber density, as previously reported [80–82]. Additionally, increased KTN concentration decreased hydrogel porosity, although not as significantly as the increase in collagen concentration, likely due to the much higher molecular weight (MW) of collagen (115–300 kDa) compared to KTN (40–60 kDa) [83]. The addition of KTN increased the fiber width of 7 mg/ml hydrogels with high counts of fibers with increased fiber width. Since KTN contains thiol groups of cysteine residues that form disulfide crosslinks spontaneously [84], KTN may have provided additional reinforcement and bundling of collagen fibers or a form of weak intermolecular bonding such as hydrogen bonding between collagen and KTN [85]. The increase in KTN also caused the fiber orientation to be more aligned. This fiber structure alignment has not been previously reported in KTN blend hydrogels, in which most fiber structures of KTN-blend hydrogels appeared amorphous [84,86,87]. This increase in fiber alignment may lead to increased spreading of cells, particularly fibroblasts, as reported in previous studies [88,89]. In addition, keratin is one of the few intermediate filament-forming proteins with a natural propensity to form fibers [83]. When extracted from human hair, this property is still present, which may drive the process of this fiber formation and an increase in fiber alignment.

Based on rheological analysis of the hydrogels, the measured G corresponded well to the previous studies of collagen hydrogels using similar methods [90–93]. Knapp et al. reported similar *G*′ values for 3 mg/ml collagen hydrogels of 10–50 Pa with identical testing parameters, similar to our study [91]. Other studies have reported a 3.5- to 6.17-fold increase in *G*′ when collagen and Matrigel matrix concentration was increased from 3 to 7 or 4 to 8 mg/ml [92,93]. Our studies showed that an increase from 3 to 7 mg/ml collagen rather than an increase in KTN concentration had a similar increase in storage modulus, with 4.89-fold increase. A previous study reported that keratin hydrogels had a similar storage modulus to a 10-fold lower concentration collagen hydrogel [94], indicating that higher keratin concentration does not contribute to higher stiffness. The significant effect of collagen, rather than KTN, increasing the storage modulus was most likely due to the higher MW of collagen than KTN, leading to a higher degree of chain entanglements and lower chain diffusion within the hydrogel [95–97]. However, results demonstrate an increasing trend in storage modulus from the entire frequency sweep of 0.1–15 Hz as KTN concentration increases. In addition, *G*′′ is lower than *G*′ for all samples and never crosses, indicating that the hydrogels are viscoelastic and crosslinked into a strong hydrogel [98].

Although collagen is an excellent representative ECM for 3D culture, it has been shown to be thermally unstable at temperatures between 38 and 45 °C [37–39], making collagen impractical for studies focusing on the effect of heat treatment or hyperthermia on cell behavior and their interaction with the surrounding ECM. Previous studies employing DSC have shown that collagen starts to denature at 42 °C and is even unstable at body temperature (37 °C) [30,39,41,99]. Tuttolomondo et al. demonstrated that collagen hydrogels tested through DSC showed a peak *T*_m_ of 55 °C, corresponding to the collagen triple helix breaking [100]. On the other hand, KTN has been shown to have a peak *T*_m_ up to 240 °C. Therefore, we hypothesized that the addition of KTN would increase the thermal stability of the composite C/KTN hydrogel. Our study shows that 3 mg/ml and 7 mg/ml collagen hydrogels had a peak *T*_m_ of 51 °C. The onset temperature or the *T*_1/2_ which occurs when the dip of the peak starts in the plot, is at a lower temperature than the peak or *T*_m_ which is the average temperature of melting. This onset temperature explains the instability of collagen at a lower melting temperature of 38–45 °C when compared to the actual peak *T*_m_. This *T*_m_ peak is due to the breaking of bonds and the change in conformation of the tertiary structure [101]. KTN’s inter- and intra-molecular disulfide bonds of cysteine are responsible for greater stability than collagen [85]. However, this disulfide bond is difficult to break in the temperature range used in the DSC experiment. Therefore, this denaturation peak may correspond to the disassembling of collagen and KTN fiber networks in which they can establish intra-molecular interactions. The added KTN may increase the chain entanglements with the collagen possibly leading to higher stability and a slight increase in *T*_m_. Our results indicate a trend in increasing denaturing temperature with greater KTN, in which KTN may act as a secondary crosslinker and stabilize the collagen hydrogels produced with primary crosslinking [60]. One study by Balaji et al. developed composite scaffolds made of bovine collagen, and KTN extracted from horn meal and characterized their thermal properties with DSC using dry scaffolds [50]. Although we were unable to directly compare our results with this study since we tested wet hydrogels, Balaji et al. observed an increase in *T*_m_ when compared to pure collagen hydrogels [50]. Another study conducted by Vu et al. created a silk fibroin-wool keratin hydrogel blend and found that blended hydrogels had higher degradation peaks measured by DSC than the pure counterpart [102]. This may be due to the interconnected networks of collagen and KTN at the nanoscale, providing reinforcement and higher thermal stability.

The addition of KTN made the gels more thermally stable, as evidenced by higher normalized weight in C/KTN hydrogels than pure collagen, while also maintaining its shape. Previous studies have used other materials such as sodium alginate to mimic tissue for thermal ablation cancer treatment because it can withstand temperatures up to 100 °C [56,57]. Other studies have used food-grade carbohydrates or chitosan to improve thermal properties for food processing [49,103]. However, these non-native materials, specifically alginate, are not cell-friendly and do not contain specific binding motifs that cell receptors can recognize [46,104]. In most cases, these materials require modifications by crosslinking cell adhesive peptides sequences such as arginylglycylaspartic acid (RGD), or combining with natural and native polymers such as collagen [27,45,49,104]. Therefore, KTN is a more attractive biomaterial to be used since it is present in the human body and contains specific binding motifs for cell attachment and proliferation without high degradation, as shown by our current results.

We demonstrated the ability of the C/KTN hydrogels as an ECM to promote cell growth and proliferation for 3D *in vitro* modeling. To mimic healthy tissue, we used 3 mg/ml hydrogels and NHDFs due to the prevalence of fibroblast in all tissues throughout the human connective tissue, specifically skin [23–26,63]. As a representative diseased tissue such as cancer, MDA-MB-231 breast cancer cells were seeded in stiffer, 7 mg/ml hydrogels [15,23,25,26,63,64,69]. Our results showed KTN had no cellular cytotoxic effect when added at lower concentrations when compared to pure collagen hydrogels. Lower viability and metabolic activity were observed in both MDA-MB-231 breast cancer cells and NHDFs (low seeding) in 20/80 C/KTN hydrogels. Although cell spreading and increased AR was observed for MDA-MB-231 breast cancer cells and NHDFs seeded in 20/80 C/KTN hydrogels, they may have initially had difficulty spreading and proliferating due to the increased protein concentration and matrix density, delaying their growth. The presence of a lower concentration of KTN, specifically for 50/50 and in some cases 30/70 C/KTN hydrogels, may have initially promoted cell attachment and proliferation due to cell binding motifs including leucine–aspartic acid (LDV) and glutamic acid–aspartic acid–serine (EDS), which can support cellular attachment and proliferation [54,55,105]. A previous study showed that keratin hydrogels encapsulating murine fibroblasts had similar cell viability and proliferation to those seeded in collagen hydrogels [106]. Another study comparing 10 mg/ml keratin and 1 mg/ml collagen hydrogels saw lower and delayed proliferation and viability with murine fibroblasts seeded on keratin hydrogels [94], possibly due to the higher concentration of protein of the keratin hydrogel. Similar results were observed in our study with 50/50 and 30/70 C/KTN hydrogels in which they had similar viability to pure collagen hydrogels.

Hydrogels seeded at 0.5 × 10^6^/ml had no observable NHDF growth over time, possibly due to spatial constraints and crowding preventing cell proliferation [107]. When seeded at lower density, however, NHDFs in all hydrogels except 20/80 C/KTN hydrogels expressed significant growth over seven days with significantly similar growth compared to 100% collagen. Also, NHDFs seeded in 50/50, and 30/70 C/KTN hydrogels resulted in more elongated NHDFs compared to 100% collagen at day one as demonstrated by a significantly higher AR. As the presence of KTN resulted in an ECM structure of aligned and bundled fibers, whereas pure collagen hydrogels were more disorganized, this may have caused the NHDFs to spread more throughout the C/KTN hydrogels, along the aligned and thicker fibers. A previous study has demonstrated that cells seeded on top of KTN hydrogels displayed morphology characterized by spreading, whereas cells grown on top of collagen were less spread [108]. Previous studies have shown that inducing fiber alignment by numerous methods, including electrospinning, creates an organized network of fibers similar to what is observed in native tissue [88,89]. Unfortunately, harsh solvents and crosslinkers are used in most cases of electrospinning, which can leave behind residual chemicals that may be toxic to the cells encapsulated within the biomaterial. Keratin on the other hand, contains binding motifs such as leucine–aspartic acid-valine (LDV), which supports cell attachment and subsequent spreading [54,55]. By using a natural crosslinker biomaterial such as keratin, toxic effects of crosslinkers can be avoided at low concentrations but still increase directionality and alignment of the fibers. Although cell viability of C/KTN hydrogels is not significantly different from that of pure collagen, slight increases in viability and metabolic activity of cells seeded in 50/50 C/KTN hydrogels reflect the elongation and spreading of cells promoted by KTN. However, this cannot be confirmed without further SEM analysis in cellular gels.

## 5. Conclusion

We developed C/KTN hydrogels and tested their potential for use in thermal stress, 3D *in vitro* studies by studying their physical, thermal and biological characteristics. Our study demonstrated that the increase in KTN altered the ECM structure by aligning the fibers in uniform orientation and slightly increased the storage modulus. More importantly, an increase in KTN concentration increased the thermal stability of the hydrogels, allowing them to maintain their shape for up to 1 h at hyperthermic temperature, reaching 47 °C. In addition, 50/50 and 30/70 C/KTN hydrogels promoted cell growth compared to 100% collagen hydrogels without having any significant adverse effects. Specifically, 50/50 C/KTN hydrogels had similar or even higher viability while maintaining higher thermal stability than pure collagen hydrogels. In future studies, this type of composite hydrogel may be used as a potential ECM for 3D *in vitro* models for thermal stress studies, including hyperthermia treatment of 3D *in vitro* cancer models or other heat shock treatments to investigate potential ablation therapies.

## Supplementary Material

S2 NHDF viability

S1 Porosity Data

## Figures and Tables

**Table 1.** KTN concentration and total protein concentration for each type of hydrogel.

Hydrogel	KTN content (3 mg/ml collagen)	KTN content (7 mg/ml collagen)
100% collagen	0 mg/ml KTN, 0.3 wt%/vol%	0 mg/ml KTN, 0.7 wt%/vol%
50/50 C/KTN	3.0 mg/ml KTN, 0.6 wt%/vol%	7.0 mg/ml KTN, 1.4 wt%/vol%
30/70 C/KTN	7.0 mg/ml KTN, 1.0 wt%/vol%	16.3 mg/ml KTN, 2.3 wt%/vol%
20/80 C/KTN	12.0 mg/ml KTN, 1.5 wt%/vol%	28.0 mg/ml KTN, 3.5 wt%/vol%

Concentration of collagen stays constant.
