# Supplementary figures and images for: Collagen/kerateine multi-protein hydrogels as a thermally stable extracellular matrix for 3D in vitro models
Source: Int J Hyperthermia. Author manuscript; Available in PMC 2023 Sep 27. (PMC10523628; doi:10.1080/02656736.2021.1930202)

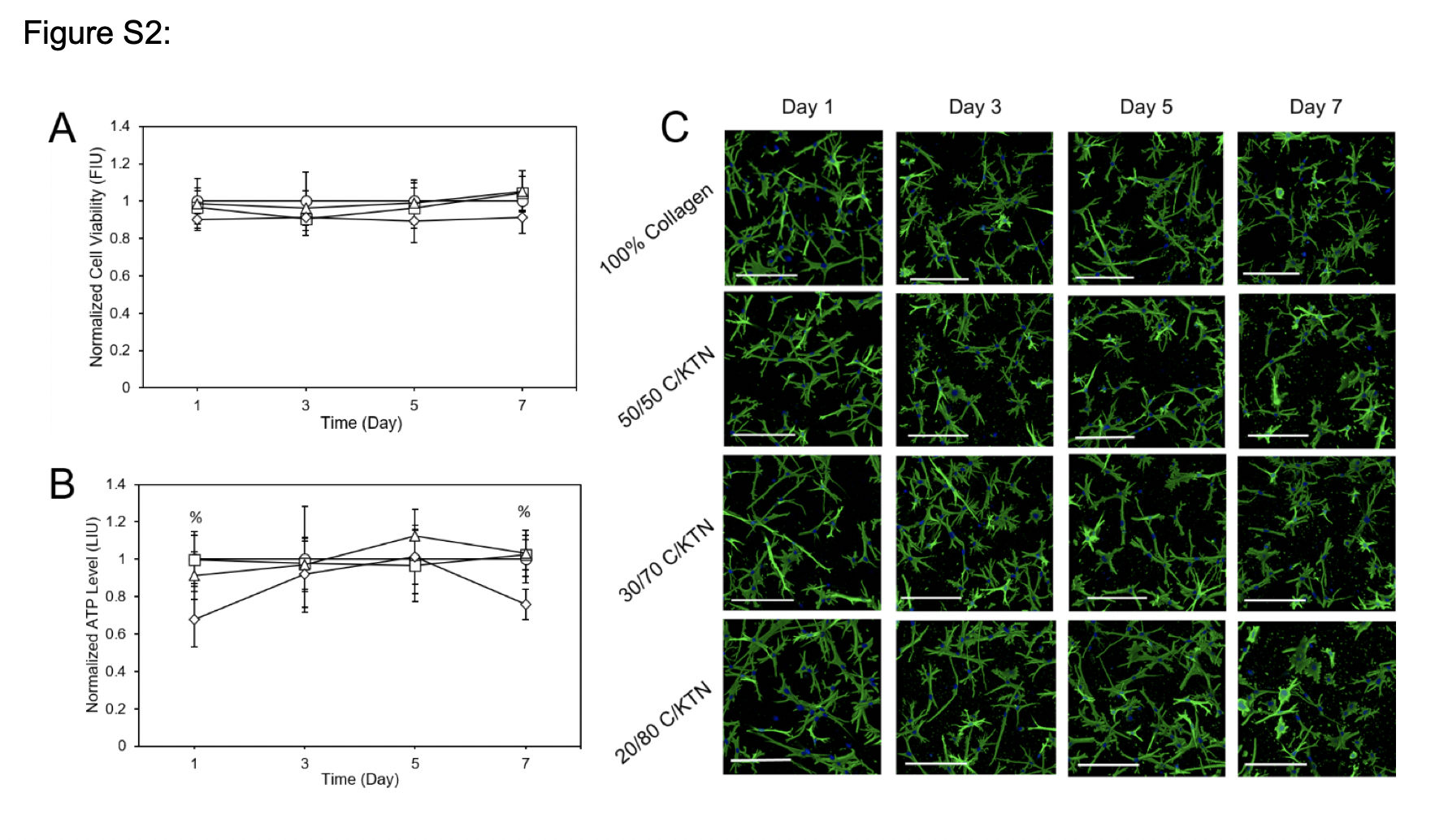

Supplement: S2 NHDF viability [file NIHMS1932534-supplement-S2_NHDF_viability.png]

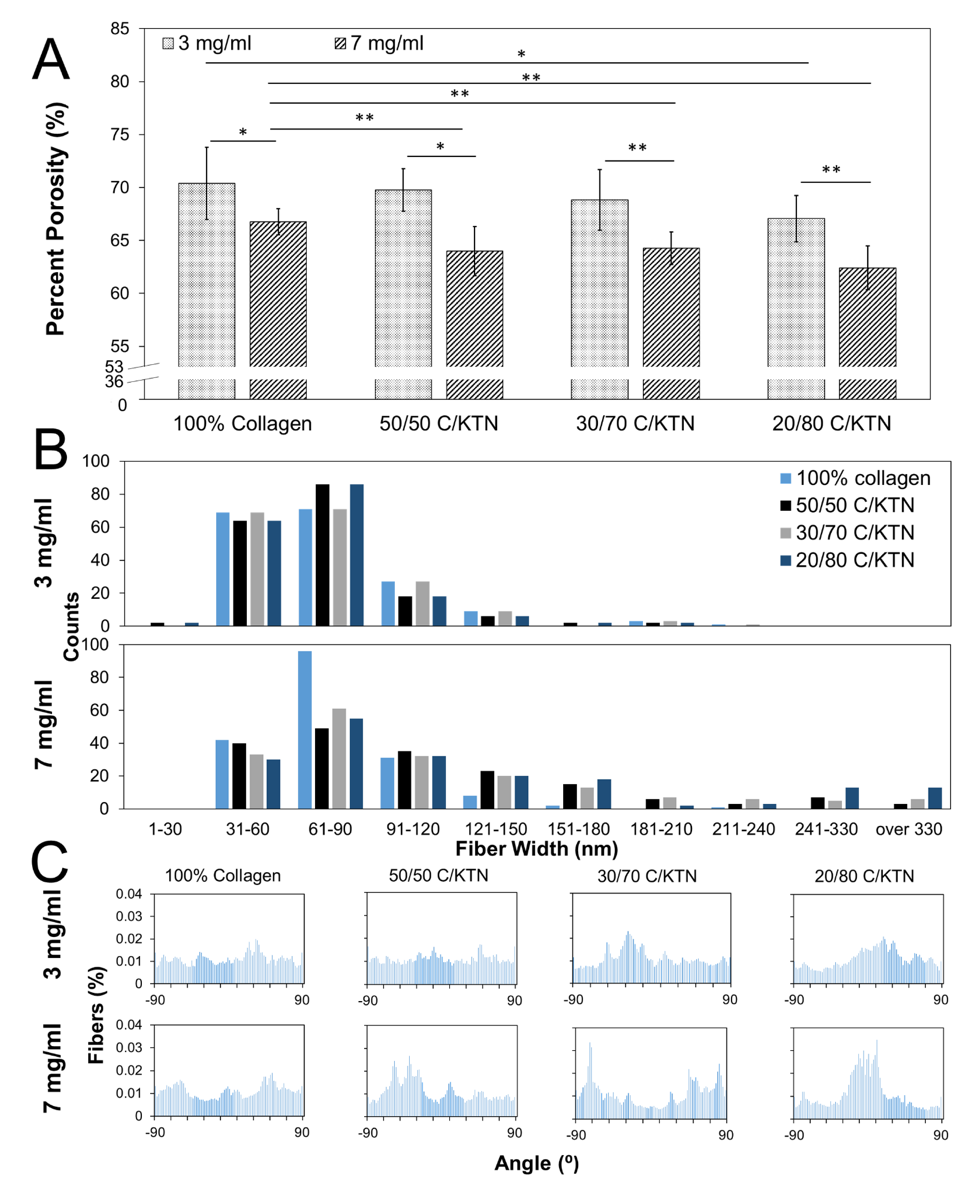

Supplement: S1 Porosity Data [file NIHMS1932534-supplement-S1_Porosity_Data.png]
